# Microencapsulation of bioactive compounds from peanut residues: stability study and application in yogurt

**DOI:** 10.1002/jsfa.70698

**Published:** 2026-05-03

**Authors:** Ana Carolina Tucumantel Ribeiro, Victor Waldemar Pinto, Jaqueline LS Cavalcante, Nathalia Almeida Costa, Bárbara M Lepaus, Juliana A Macedo, Patrícia Blumer ZR Sá, Sílvia Cristina Sobottka Rolim de Moura

**Affiliations:** ^1^ Institute of Food Technology – ITAL – Postgraduate Program São Paulo Brazil; ^2^ Institute of Food Technology – ITAL São Paulo Brazil; ^3^ Food Engineering Faculty, State University of Campinas – UNICAMP São Paulo Brazil

**Keywords:** dairy food, ionic gelation, polyphenols, spray drying, waste

## Abstract

**BACKGROUND:**

Peanut skin, a byproduct of peanut processing, is a rich source of phenolic compounds (520.65 mgGAE (100 g)^−1^ d.b.) with strong antioxidant capacity, but which remains underutilized. The direct incorporation of phenolic extracts into dairy products is challenging, due to their instability during processing and storage and possible interactions that may affect product quality. Encapsulation techniques can improve the stability and functionality of these bioactive compounds. This study evaluated peanut skin as a sustainable source of phenolics and compared two encapsulation methods – spray drying and ionic gelation – to stabilize its extract prior to incorporation into yogurt, with quality monitored over 42 days of storage.

**RESULTS:**

Spray drying with maltodextrin and ionic gelation using pectin–starch microparticles crosslinked with calcium chloride were applied as stabilization strategies. Both methods achieved high encapsulation efficiency (90–95%) and similar phenolic concentrations, although wet ionic gelation microparticles presented a more intense reddish color. During storage, the microparticles effectively preserved phenolic compounds, antioxidant activity, and color stability. When incorporated into yogurt, phenolic content reached 18.87 mgGAE g^−1^ d.b. for spray‐dried particles and 18.20 mgGAE g^−1^ d.b. for wet particles. Antioxidant activity remained around 75%, while color variation was minimal (Δ*E* < 3.56). No significant changes were observed in acidity, proximate composition, or microbiological quality.

**CONCLUSION:**

Peanut skin can be converted into a stable, value‐added functional ingredient for yogurt. Both encapsulation techniques preserved the phenolic compounds and product quality during storage, representing a scalable and sustainable strategy to enhance the nutritional value of dairy products while reducing agro‐industrial waste. © 2026 The Author(s). *Journal of the Science of Food and Agriculture* published by John Wiley & Sons Ltd on behalf of Society of Chemical Industry.

## INTRODUCTION

Agro‐industrial residues have attracted attention due to their valuable composition, including carbohydrates, proteins, and antioxidant compounds, offering low‐cost raw materials with commercial potential.^1^ Phenolic compounds are known for their ability to neutralize reactive oxygen species and prevent lifestyle‐related diseases, although their effectiveness depends on intake, digestion, and bioavailability.^2^

Peanut skin, representing about 3% of peanut mass and generating approximately 0.93 million metric tons annually worldwide, is largely discarded despite being rich in proteins (12%), lipids (16%), carbohydrates (72%), and polyphenols (140–150 mg g^−1^), particularly catechin and proanthocyanidins with strong antioxidant activity.^3^, ^4^, ^5^ Its phenolic fraction has important nutritional and pharmaceutical value, as well as an increasing application as a functional ingredient.^6^

Microencapsulation is an effective strategy to protect bioactive compounds, improving their stability and expanding their applications.^7^ In the food industry, such strategy prevents degradation, masks undesirable flavors, reduces hygroscopicity, and enables incorporation into dry systems.^8^ Several authors have raised the importance and need for the use of edible and biodegradable films and various biopolymers (such as maltodextrin, glutenin, or surfactants as a medium of extraction) for the production of films/coatings for microencapsulation of bioactive compounds (antioxidants and natural preservatives).^9^, ^10^, ^11^ Among several existing microencapsulation methods, ionic gelation is a simple and low‐cost technique that provides acceptable protection of bioactive compounds,^12^, ^13^ while spray drying is widely used for heat‐sensitive compounds due to its efficiency, simplicity, and production of high‐quality powders.^14^

The functional food market, particularly of dairy products, continues to grow as consumers seek health‐promoting foods.^15^ Although yogurt is recognized for its probiotic and bioactive peptide benefits,^16^, ^17^ incorporating additional bioactive compounds is challenging due to sensory and formulation constraints.^18^, ^19^

Therefore, the study reported here aimed to encapsulate peanut skin extract obtained from agro‐industrial waste, as well as to evaluate the stability of the microencapsulated extract, using two methods: ionic gelation and spray drying. Also, the study intended to investigate peanut skin extract application in traditional yogurt to improve functional properties, simultaneously promoting the sustainable valorization of waste.

## MATERIALS AND METHODS

### Materials

Runner variety peanut skin, classified as type 1, was supplied by Cooperativa Coplana (Guariba – SP) in the form of pellets. The encapsulation materials were low methoxylation (BTM) amidated pectin GENU® 104‐AS‐Z (CP Kelco, Limeira/Brazil), food‐grade calcium chloride (Dinâmica, Diadema/Brazil), Maizena cornstarch, and maltodextrin (Morrex 1910 D10). The following analytical‐grade (PA) reagents with purity above 99% were used for the evaluations: ethyl alcohol, acetone, EDTA, potassium chloride, sodium carbonate, sodium acetate trihydrate, 0.06 mmol L^−1^ DPPH solution, ABTS solution, potassium persulfate, 2 nmol L^−1^ Trolox solution, gallic acid, Folin–Ciocalteu reagent, potassium bromide, potassium chloride, hydrochloric acid, monobasic potassium phosphate, and sodium hydroxide (5 mol L^−1^). For the production of yogurt: skimmed milk powder (La Sereníssima®, Bragança Paulista/Brazil) and thermophilic starter culture – YCE128A (Sacco System, Sousas/Brazil). For the production of regular or traditional yogurts: pasteurized type A skimmed light milk A Xandô® (Laticínios Xandô, Araras/ Brazil) (9 kg m^−3^ of total solids), of which 2% were used, composed of the microorganisms *Streptococcus thermophilus* and *Lactobacillus delbrueckii* ssp. *bulgaricus* (LYOFAST Y 450B (Sacco System/Italy).

### Methods

#### Extract production

The phenolic extract was produced by a partner company, Centroflora Inova (Campinas – SP). In order to obtain the extracts, the residue was treated with a 50% (v/v) alcoholic solution in a proportion of 1:20 (w/v). Thereafter, the mixture was kept in ultrasound equipment (Ultronique, Model Q9.5/40A) for 30 min under heating (25–30 °C). After this period, the extract was filtered in a filter bag with a 100 μm mesh and sent to a rotary evaporator (R‐100, BUCHI Brasil Ltda, Valinhos/Brazil) to recover the solvent.

The extract was characterized using the methods described in the following subsections.

##### Total phenolic compound content

Total phenolic compound content was determined according to the spectroscopic method of Folin–Ciocalteau, used by Erkan‐Koç *et al*.^20^ Sample and standard curve readings (*n* = 3) were conducted using a UV–visible spectrophotometer (Cary 60 MY13110012, Agilent Technologies, USA) at a wavelength of 750 nm. The results were expressed as mgGAE (100 g)^−1^, where mgGAE is milligrams of gallic acid equivalent.

##### Moisture content

The moisture content was determined gravimetrically (drying oven, Quimis Q319V2, Diadema, Brazil) by drying at 70 °C without vacuum for 24 h, followed by vacuum drying for another 24 h. The analysis was performed (*n* = 3) with approximately 10^−2^ kg of sample.^21^

##### Identification and quantification of phenolic compounds by high‐performance liquid chromatography (HPLC)

For phenolic compound extraction from peanut skin liquid extract, 2 × 10^−3^ kg of sample was homogenized with 2 × 10^−6^ m^3^ of H_2_O. This solution was quickly homogenized by vortexing and then centrifuged (166.7 Hz, 15 min, 4 °C). The supernatant was collected and a new dilution was performed (5 × 10^−7^ m^3^ of supernatant +1.5 × 10^−6^ m^3^ of H_2_O). This solution was quickly homogenized by vortexing and then centrifuged in Amicon® Ultra 10k tubes (83.3 Hz, 60 min, 4 °C). The lower fraction was collected, filtered (0.45 μm filter, PVDF), and injected into the HPLC system.

The phenolic compounds present in the extract were analyzed by HPLC, according to the methodology described by Costa *et al*.,^22^ using a Dionex Ultimate 3000 HPLC system (Dreiech, Germany) coupled with a diode array detector and C18 column (3 μm, 120 Å, 4.6 × 10^−3^ m × 15 × 10^−2^ m) for separation at 30 °C. Mobile phases (A) H_2_O–formic acid (99.9:0.1 v/v) and (B) methanol–formic acid (99.9:0.1 v/v) were used in gradient mode: 92% A (0–300 s), 92% A (300–780 s), 85% A (780–2700 s), 75% A (2700–4020 s), 57% A (4020–4620 s), 50% A (4620–5700 s), 35% A (5700–5820 s), and 92% A (97–107 min). The solvent flow rate was 8.3 × 10^−9^ m^3^ s^−1^. The standards used for identification and quantification of the compounds were from Extrasynthese: catechin (0976S), epicatechin (0977S), epicatechin gallate (0978S), procyanidin B1 (983); and from Sigma: *p*‐coumaric acid (C9008‐1G), epigallocatechin gallate (E4143), and procyanidin B2 (42157). All compounds were quantified at a wavelength of 280 nm, except for *p*‐coumaric acid, which was quantified at 320 nm. The results were expressed as 10^−9^ g kg^−1^ of liquid extract.

#### Microencapsulation

**Figure 1:**
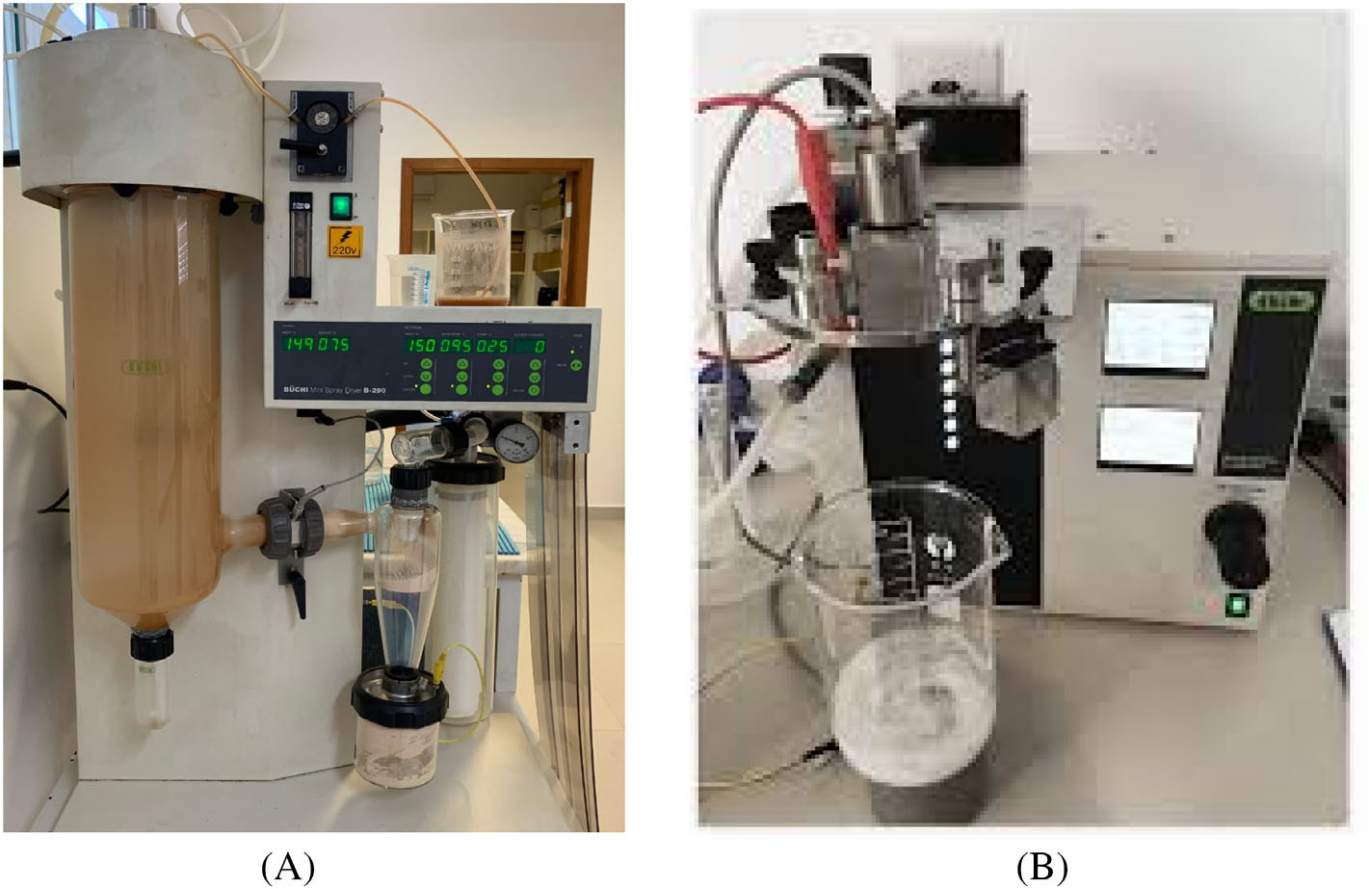
(A) Spray dryer (Buchi B‐290) for DP production. (B) Encapsulator (Buchi B‐390) for WP production.

Dry microparticles (DP) were produced (1) by drying in a spray dryer (B‐290, BUCHI Brasil Ltda, Valinhos/Brazil) (Fig. 1(A)) and wet microparticles (WP) were produced (2) by ionic gelation using an Encapsulator B390 (BUCHI Brasil Ltda, Valinhos/Brazil) (Fig. 1(B)), followed by absorption of the peanut skin extract by the microparticles.

Microencapsulation was updated to include the experimental design for obtaining microparticles^23^; the methodologies were based on those of Budin *et al*.^24^ and Moura *et al*.^25^

For the ionic gelation method, the independent variables were vibration frequency (100 to 2200 Hz) and electrode voltage (400 to 2000 V), with the nozzle diameter set at 450 μm. The distance from the nozzle to the surface of the calcium solution was set at 10 cm. The particle curing time in the calcium solution was 15 min, under agitation.

For the spray drying method, the independent variables were drying air temperature (160–220 °C) and feed flow rate (8.3–25 × 10^−8^ m^3^ s^−1^), with the nozzle diameter set at 0.7 × 10^−3^ m. Dependent variables for both methods were the mean diameter, size distribution, encapsulation efficiency (EE), microscopy, and color.

The best process conditions were used for both DP and WP. For DP, 500 × 10^−3^ kg of extract + 27.86 × 10^−3^ kg of maltodextrin, the process conditions were: inlet temperature (149 °C) leading to (75 °C), air pressure (25%), and a feed rate of 95% (5.3 × 10^−8^ m^3^ s^−1^). WP were obtained by ionic gelation followed by absorption, based on Silveira *et al*.^8^ They were produced in Encapsulator equipment, generating microparticles (pectin + starch) without active, which in turn formed a solution formulated with 70% pectin 2% and 30% starch 0.85%. The best process conditions were: nozzle 300 μm, *P* = 15.4 kPa, frequency = 100 Hz, voltage = 250 V. Subsequently, the microparticles were submerged in the peanut skin extract in a proportion of 1 (microparticles) to 2 (extract), for 86.4 × 10^3^ s.

The analysis methods used to characterize the microparticles were as described in the following subsections.

##### Average diameter and size distribution

The samples had their average diameters and size distribution determined using a laser light scattering analyzer (LA‐900, Horiba Instruments, Japan) by light scattering using a liquid dispersion module. The average diameter was determined based on the average diameter of a sphere of the same volume (De Brouckere diameter D[4.3]), calculated according to Eqn (1):

(1) $$D\left[4,3\right]=\frac{\sum {n_{i}d_{i}}^{4}}{\sum {n_{i}d_{i}}^{3}}$$

where *d*_*i*_ is the diameter of the microparticles and *n*_*i*_ is the number of microparticles.

The polydispersity index (ID) was calculated according to Santos *et al*.,^26^ using Eqn (2):

(2) $$\mathrm{ID}=\frac{d_{90}-d_{10}}{d_{50}}$$

where *d*_10_, *d*_50_, and *d*_90_ are the diameters at 10%, 50%, and 90% of the accumulated volume, respectively, that is, *d*_90_–*d*_10_ is the data range and *d*_50_ is the average diameter.

##### Total phenolic compounds

The method used was as described in Total phenolic compound content section. The active ingredient was extracted from the wet microparticles by dissolving them in EDTA and extracting them with 70% ethanol and acetone (70%), alternately.^25^ The active ingredient was extracted from the dry microparticles by diluting them in water (1:20) and extracting them with 70% ethanol.

##### Antioxidant activity

The antioxidant activity was determined using the DPPH and ABTS methods according to the methodology described in Jiménez‐Zamora *et al*.^27^ Absorbance readings (*n* = 3) were performed with a UV–visible spectrophotometer (Cary 60 MY13110012, Agilent Technologies, USA) at a wavelength of 515 nm (DPPH) and 734 nm (ABTS). The results were expressed a μmol TE g^−1^ (Trolox equivalent).

##### Encapsulation efficiency

EE indicates the amount of active ingredient (total phenolic compounds, TPC) that was effectively retained in the microparticle structure after processing^8^:

(3) $$\mathrm{EE}\;\left(\%\right)=\frac{\mathrm{TPC}\;\text{in microparticles}}{\mathrm{TPC}\;\text{in the extract added to the microparticles}}\times 100$$

#### Stability of microparticles

The produced WP were stored in peanut skin extract and the DP were stored in a poly(ethylene terephthalate) packaging, put in a refrigerator, and protected from light for 42 days.

##### Instrumental color

The parameters L*, a*, b*, and chroma $\left(\sqrt{a{*}^{2}+b{*}^{2}}\right)$ of the color (*n* = 9) (Chromameter CR‐400, Konica‐Minolta Sensing, Osaka, Japan) were evaluated during the shelf‐life monitoring.^28^

##### Phenolic compound content

This was determined according to the methodology described Total phenolic compounds section, following the methodology mentioned in Total phenolic compound content section.

##### Antioxidant activity

This was determined in accordance with Antioxidant activity section.

#### Yogurt production

Two samples of traditional yogurt were made in the pilot plant at Tecnolat/ITAL. For the production of regular or traditional yogurts, 3.3% skimmed milk powder (La Sereníssima®, Bragança Paulista/Brazil) was added to correct the solids content. The mixture of skimmed milk (La Serenissima®) and milk powder (Laticínios Xandô, Araras/Brazil) was heat treated at 90 °C for 5 min, followed by cooling to fermentation temperature (around 43 °C), yeast inoculation, and incubation in BOD (biochemical oxygen demand) at the same temperature until reaching a pH value of 4.6. The sample was then cooled to 25–30 °C, homogenized manually for 120 s, bottled, and cooled. Then, 10% of WP and 0.6% of DP were added, aiming for similarity in the content of phenolic compounds; subsequently, the packages were stored under refrigeration (5 °C).

The dry particle yogurt (DPY) and wet particle yogurt (WPY) (*n* = 3) were characterized in terms of:

- Microbiology – total lactic bacteria, based on Compendium APHA.^29^
- Centesimal composition – moisture and volatiles, based on AOAC^21^ official method 990.20.
- Ash, based on AOAC^21^ official method 945.46.
- Total fat, based on IAL,^30^ method 034B.
- Protein, based on IAL,^30^ method 037.
- Total carbohydrates, calculated by difference: 100 − (g kg^−1^ moisture + g kg^−1^ ash + g kg^−1^ total fat + g kg^−1^ protein).
- Energy value – based on Passmore *et al*.^31^
- Instrumental color (same as in Instrumental color section).
- Phenolic compounds (same as in Phenolic compound content section).
- Antioxidant activity – DPPH (same as in Antioxidant activity section).

#### Study of yogurt stability

The freshly processed yogurts were packaged in polyethylene containers and stored at 5 ± 2 °C. The analyses were carried out during 42 days of storage. The methods used were as follows.

##### Total phenolic compounds

The quantification of the recovered active ingredient was performed in accordance with Phenolic compound content section.

##### Antioxidant activity

The quantification of the recovered active ingredient was performed in accordance with Antioxidant activity section.

##### Color analysis

This was performed in accordance with Instrumental color section. Additionally, the parameter ∆*E* (total color variation) was obtained by using Eqn (4)^28^:

(4) $$∆E=\sqrt{{\left(∆L^{*}\right)}^{2}+{\left(∆a^{*}\right)}^{2}+{\left(∆b^{*}\right)}^{2}}$$

##### Evaluation of pH

The pH evaluation was performed using a potentiometer (Digimed, model DM 20, Brazil) according to the methodology described in IAL^30^ (*n* = 3).

##### Total acidity

The total acidity evaluation was performed in accordance with AOAC no. 942.15B^21^ (*n* = 3).

#### Statistical methods

Statistical analysis was performed using Statistica® version 12 (StatSoft Inc., Tulsa, EUA) with a one‐way analysis of variance and Tukey's test. The results were presented as mean ± SD. The differences were considered statistically significant if *P* < 0.05.

## RESULTS AND DISCUSSION

### Extract characterization

The extract was characterized in terms of the total amount of phenolic compounds, obtaining the results expressed on a wet and dry basis in Table 1.

**Table 1:** Total phenolic compounds of peanut skin extract

Sample	Total phenolic compounds
Peanut skin extract	520.65 ± 21.83 d.b.
	2377.76 ± 322.49 w.b.

*Note:* Units: (mgGAE g^−1^) d.b. and (mgGAE (100 g)^−1^) w.b.; extract moisture: 95.51 ± 1.84%.

Munekata^32^ achieved a result of 32.6 ± 0.7 mg GAE g^−1^ peanut skin. In a parallel with the extract used in this study, the values obtained here were higher; Calomeni^33^ determined 42.88 ± 4.39 mg of phenolics per gram of extract, which is also a smaller quantity. Costa *et al*.,^22^ when evaluating peanut skin extract, found a phenolic compound content corresponding to 401.08 ± 21.31 mg GAE g^−1^ of extract, also a smaller quantity.

The different extracts can cause this difference in phenolic content values, as a consequence of time, type of extraction, and temperature, among other factors, which alter the quantity of bioactive compounds. Another factor that had an impact on the quantity of phenolics was the lack of control over the temperature and pressure of the vacuum pan used in the extract production.

Identification and partial quantification of phenolic compounds from peanut skin extract by HPLC are presented below. The values are expressed as 10^−9^ g kg^−1^ of liquid extract. Only one sample preparation was performed due to the high cost of the Amicon filter (*n* = 1). It is worth noting that this is a partial quantification since it represents the portion of the sample with molecular weight below 10 kDa.

Compounds: *p*‐coumaric acid, 2.1 × 10^−9^ g kg^−1^; catechin, 26.75 × 10^−9^ g kg^−1^; epicatechin, 236.38 × 10^−9^ g kg^−1^; epicatechin gallate, 28.90 × 10^−9^ g kg^−1^; epigallocatechin gallate, 5.97 × 10^−9^ g kg^−1^; procyanidin B1, 7.67 × 10^−9^ g kg^−1^; procyanidin B2, 31.59 × 10^−9^ g kg^−1^.

According to Costa *et al*.,^22^ at the highest levels of peanut shell extract, compounds such as epicatechin and epicatechin gallate were the main phenolic compounds detected, followed by catechin and procyanidins. The same results were observed in the present study.

### Production of microparticles

#### Dried microparticles

**Figure 2:**
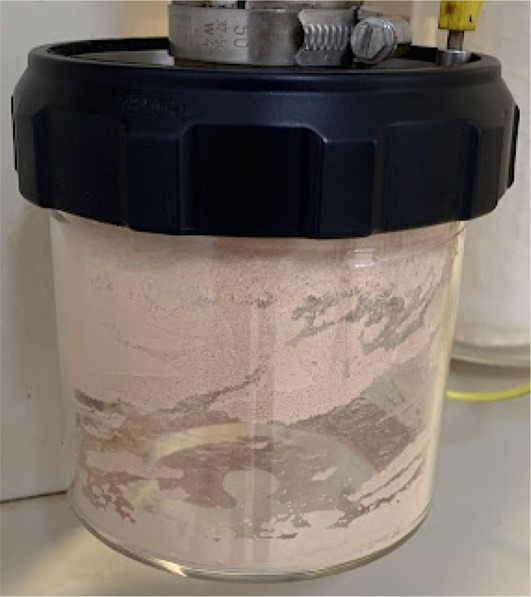
Microparticle deposit from the spray dryer with peanut skin extract microparticles.

The microparticles produced using a spray dryer are shown in Fig. 2. As a characteristic of microparticles obtained by using this method, the final appearance is a fine, light pink powder.

#### Wet microparticles

**Figure 3:**
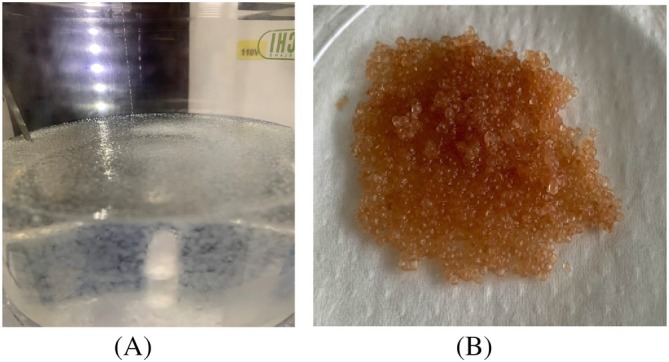
Wet microparticles (A) before and (B) after immersion in peanut skin extract.

After being produced by ionic gelation (Fig. 3(A)), the pectin and starch microparticles were immersed in the extract for 24 h, resulting in what is shown in Fig. 3(B). The final appearance of these microparticles is that of small balls of shiny gel and a dark red, almost brown color, very similar to peanut skin extract color.

### Characterization of microparticles

The microparticles were characterized as total phenolic compounds, resulting in the amounts presented in Table 2.

**Table 2:** Total phenolic compounds of peanut skin extract microparticles on dry and wet basis

Microparticle	Total phenolic compounds
DP	266.84a ± 22.45 d.b. / 26 016.19 ± 2189.53 w.b.
WP	254.56a ± 2.57 d.b. / 2138.36 ± 21.59 w.b.

*Note:* Results expressed as mean ± standard deviation, *n* = 3; means followed by different lowercase letters differ statistically at the 5% error level by Tukey's test (*P* ≤ 0.05). Units: (mgGAE g ^−1^) d.b. and (mgGAE (100 g)^−1^) w.b. DPY, Dry particles. WPY, Wet particles.

The results showed that the microparticles obtained by the ionic gelation method followed by the absorption technique (WP) presented a similar amount (d.b.) of phenolic compounds to DP (Table 2).

WP presented high moisture of 91.60 ± 2.04, while moisture of DP was 2.49 ± 1.08 (Aw 0.375 ± 0.05); therefore, it was necessary to express the results on a dry basis for all the microparticles, in order to allow a better comparison between them. When calculating the EE, a value of 95% was obtained for DP, while WP achieved 90%. Budin *et al*.^24^ observed values of 87.86 ± 1.79 mgGAE (100 g)^−1^ on a wet basis for microparticles of yerba mate extract obtained by ionic gelation, with EE of 45%. Santos *et al*.^26^ obtained EE values of 25.76% to 54.51% in terms of phenolic compounds in the encapsulation of *pitanga* (*Eugenia uniflora* L.) extract by ionic gelation followed by fluidized bed drying. These results are well below the values found in this study, which reached a concentration of 2138.36 ± 21.59 mgGAE (100 g)^−1^ on a wet basis. In spray dryer microencapsulation, values of 90% or higher can be easily found.^9^

Romanini *et al*.^34^ produced grape pomace extract microparticles through freeze drying, determining a quantity of phenolics of 1473 ± 21 mgGAE (100 g)^−1^, reaching a larger value than that for the microparticles in this work. Gül^9^ studied the microencapsulation of royal jelly in different proportions of gum arabic and maltodextrin by using spray drying, observing a total phenolic content of 128.91 mgGAE (100 g)^−1^, which is a lower value than that for the microparticles in this work. This difference can be explained by the crops (grape and royal jelly) and methodologies (freeze drying and spray drying) for obtaining the extracts and microparticles.

If the spray dryer and ionic gelation methods are compared, the advantages of spray dryer are considered to be a rapid, continuous, simple, economic, reproducible, and easy‐to‐scale‐up method in comparison with other drying processes. Regardless of the numerous advantages, relatively high drying temperatures can damage sensitive compounds such as lycopene, *β*‐carotene, anthocyanins, vitamin C, colors, and flavors. Additionally, low product yield is reported due to loss of dry particles in the wall of the drying vessel.^35^

Ionic gelation is a simple and easy procedure, which does not require specialized equipment, high temperature, or organic solvent and it can be considered of low cost. However, one of its disadvantages is the occurrence of heterogeneous gelation of gel particles due to the diffusion mechanism, as surface gelation often occurs before core gelation, which in this case becomes a soft core. A limitation of the ionic gelation technique lies in its restricted applicability on a large scale. However, recent studies have explored the use of microfluidic devices as a promising strategy for process scale‐up (scale‐out) and the development of portable systems for on‐demand production. Further large‐scale evaluations are still required to confirm ionic gelation feasibility for industrial implementation.^36^

Hamed *et al*.^37^ produced peanut skin extract microparticles through freeze drying, determining a quantity of phenolics of 109.46 mgGAE g^−1^, reaching a lower value than that for the microparticles in this work.

The encapsulation of peanut skin extracts with maltodextrin has been proven to effectively reduce the intensity of bitterness and astringency by delaying their solubilization in the mouth by saliva. The chemical antioxidant activity of the extracts was not found to be affected by the processing, indicating that peanut skin extracts could serve as an antioxidant when added as food ingredients. One advantage of using peanut skin extracts is that these ingredients could be considered as natural sources of antioxidants.^38^

Bergesse *et al*.^39^ evaluated the microencapsulation of phenolic compounds from peanut shells by spray drying, evaluating their physicochemical properties, storage stability, and the effect against oxidative mobility in nut kernels. Microencapsulation employed 10%, 20%, and 30% maltodextrin. The product contained 950.29 mgGAE g^−1^ of total polyphenolic compounds, a value much higher than those found in this study.

The peanut skin phenolic compounds and antioxidant activity are extremely variable due to different factors, i.e. peanut species, roasting temperature and time, skin removal methods, and solvent types used for extraction.^36^

#### Instrumental color

The microparticle colors are expressed in Table 3.

**Table 3:** Evaluation of microparticle color on the L*, a*, b* scale

Microparticle	L*	a*	b*	C*
DP	81.51a ± 0.64	5.02b ± 0.03	20.25b ± 0.17	20.86b ± 0.27
WP	44.00b ± 0.76	14.26a ± 0.21	22.63a ± 0.36	26.75a ± 3.76

*Note:* Results expressed as mean ± standard deviation, *n* = 9; means followed by different lowercase letters differ statistically at the 5% error level by the Tukey test (*P* ≤ 0.05). DPY, Dry particles. WPY, Wet particles.

Analyzing the color parameters, it can be noted that WP are darker (lower L* values) than DP. As well as a redder color, it was also due to the higher values of the a* parameter in WP, while DP had lower a* values and higher L* values (lighter).

Centomo *et al*.^40^ evaluated the bioactive properties and storage stability of microcapsules obtained by coencapsulation of native whey yeast *Kluyveromyces marxianus* VM004, known for its potential probiotic properties, and peanut skin polyphenolic extract. The microcapsules presented C* values between 9.86 and 12.01, which are lower than those found in this study.

The potential for adding color to the particles obtained in the present study proved to be interesting from the point of view of application in food.

#### Microparticle size

For *D*_50_ values, sizes of 785.97 ± 20.16 and 10.39 ± 0.28 μm were observed for wet and dry microparticles, respectively, with WP presenting a size more than 75 times the size of DP. Gül^9^ studied the microencapsulation of royal jelly using spray drying and found a particle size of 619.76 nm or 0.61976 μm. This value is much lower than that for DP obtained in the present study (10.39 ± 0.28 μm). These differences may be related to the type of equipment, type of atomizer, and process conditions employed.

The observed IDs were 1.27 for WP and 1.70 for DP. These values indicate that the two microparticle systems have similar homogeneity, with considerable variation between the sizes of the same microparticles.

Depending on the type of product where these microparticles can be applied, choosing smaller or larger particles can be a strategic decision.

The choice between spray drying and ionic gelation depends on the nature of the bioactive compound, the desired particle characteristics for application in different foods, and industrial feasibility. Spray drying is generally preferred for the commercial‐scale production of stable powders, while ionic gelation is more appropriate for encapsulating highly heat‐sensitive compounds that require mild processing conditions. Spray drying is highly suitable for large‐scale food production and continuous processing, whereas in the ionic gelation technique, conventional pearl formation methods (e.g. dripping or extrusion) are slower and less suitable for high‐throughput industrial production. In some applications, combined strategies are also explored – for example, particle production via ionic gelation followed by drying or secondary coating^24^, ^26^ – to integrate the stability advantages of both approaches.

### Microparticle stability

**Figure 4:**
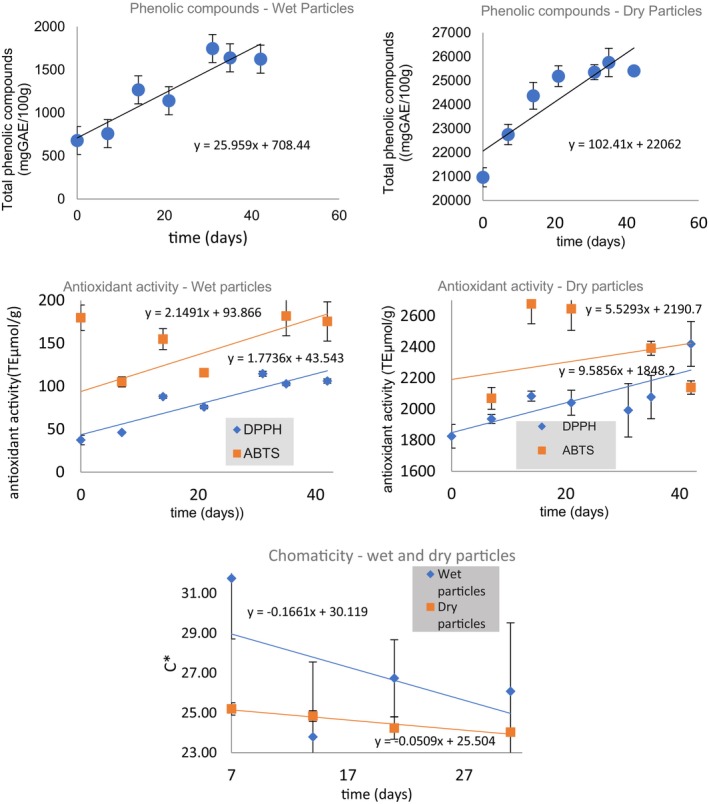
Stability of wet and dry peanut skin microparticles during storage.

The results for phenolic compounds, antioxidant activity (DPPH and ABTS), and color change of WP and DP are presented in Fig. 4.

During 42 days of storage, a difference was observed in the initial and final values for all parameters monitored, with the color in chroma as the only one showing a reduction, which indicates that the microparticles became slightly more opaque over time, especially WP. As for the amount of phenolics and antioxidant activity of both microparticle types, the values tended to stabilize or increase if the trend line is observed, which may indicate a satisfactory stability over time at controlled temperature storage.

The quantification of total phenolics in microparticles was performed using the Folin–Ciocalteau method. According to Erkan‐Koç *et al*.^20^ and Moura *et al*.,^25^ some components present in the samples have an interfering effect on this analysis and the method can only be reliable after these interfering compounds have been removed. Therefore, there may be changes in the behavior of the bioactives present in the microparticles when they are applied to food matrices.

Esparza *et al*.,^41^ when comparing the evolution of the antioxidant activity and total polyphenol content with the concentration of individual polyphenols during storage, detected a different behavior as the decrease in antioxidant capacity and total polyphenol content over time is not proportional to that of the individual phenolic compounds. This lack of correlation is in agreement with the results obtained by other authors^42^ and was attributed by Moser *et al*.^43^ to the formation of new phenolics, with equal or even improved antioxidant activities that compensate for the loss of the original phenolics.

### Characterization of yogurts with microparticles

The addition of wet or dry microparticles did not significantly alter the existing lactic bacteria (1 × 10^9^ CFU g^−1^ for both yogurts).

The microbiological identity and quality standard for yogurt (IN 46/2007, BRAZIL)^44^ establishes that the minimum total lactic bacteria count must be 10^7^ CFU g^−1^. Both samples of yogurts with microparticles presented counts of 1 × 10^9^ CFU g^−1^ after manufacture and after 42 days of storage. Thus, the produced samples had above the minimum count required even after 42 days of storage.

The phenolic compound content and antioxidant capacity achieved (Table 4) were similar for both types of yogurt (DPY and WPY).

**Table 4:** Total phenolic compounds and antioxidant activity of yogurts with and without peanut skin extract microparticles

Yogurt	Total phenolic compounds (mgGAE (100 g)^−1^)	Antioxidant activity (μmol TE g^−1^)
DPY	175.07 ± 6.89a	4.49 ± 0.26a
WPY	178.29 ± 2.29a	3.89 ± 0.35a
Without microparticles	68.22 ± 1.12b	n.d.

*Note:* Results expressed as mean ± standard deviation, *n* = 9; means followed by different lowercase letters differ statistically at the 5% error level by Tukey's test (*P* ≤ 0.05). n.d., not detectable. DPY, Dry particles yogurt. WPY, Wet particles yogurt.

Ionic gelation technique used to obtain WP has been widely used to encapsulate bioactive compounds and improve their stability in foods. According to Said *et al*.,^45^ the formation of continuous networks through calcium‐mediated ionic gelation can protect phenolic compounds, with controlled release and preservation of their antioxidant properties. This mechanism may justify the similarity observed in the levels of phenolic compounds between DPY and WPY, suggesting that encapsulation by ionic gelation may be an excellent strategy to maintain the action of added bioactives.

These results are in line with recent studies on the incorporation of bioactive compounds into yogurts in order to improve their functional and sensory properties, as observed by Ye *et al*.,^46^ who concluded that the addition of buckwheat to yogurt resulted in an increase in bioactivity and sensory acceptance of the product. There was also an influence on the physicochemical characteristics, such as acidity and viscosity of the yogurt.

The incorporation of microparticles containing peanut skin extract into yogurts resulted in a significant content of phenolic compounds, with values of 175.07 mgGAE (100 g)^−1^ w.b. or 18.87 mgGAE g^−1^ d.b. for the DPY formulation and 178.29 mgGAE (100 g)^−1^ w.b. or 18.20 mgGAE g^−1^ d.b. for WPY. These values are relevant when considering that the source used is agro‐industrial waste, highlighting the potential of peanut skin as a sustainable functional ingredient. Hamed *et al*.^37^ found similar phenolic compound values (18.80 mgGAE g^−1^) when 100 mg L^−1^ of peanut skin extract microencapsulated by freeze drying was added to yogurt. Moura *et al*.^47^ studied the application in yogurt of particles obtained by ionic gelation containing *Hibiscus sabdariffa* L. extract and obtained 5.45 ± 0.20 mgGAE (100 g)^−1^ of phenolic compounds, a value much lower than the one obtained in the present study. Unlike these raw materials, peanut skins represent an alternative for reusing a byproduct from the food industry, adding value to a residue that would otherwise be disposed of incorrectly.

The antioxidant capacity of the yogurts in this study was 4.49 μmol TE g^−1^ for DPY and 3.89 μmol TE g^−1^ for WPY, showing that peanut skin extract contributes to the antioxidant functionality of the product. Moura *et al*.^47^ found lower values in a study with *Hibiscus sabdariffa* L. microparticles (0.22 ± 0.02 μmol TE g^−1^). Jimenez‐Gonzalez *et al*.^48^ evaluated the implication in yogurt of microparticles containing *Renealmia alpinia* extract obtained by spray drying, achieving values of 1.1 mg TE 100 g^−1^. It should be noted that the method for determining antioxidant activity is different from that used in the present study and the results are presented in different units; therefore, they cannot be compared. In the present study using peanut skin, the presence of good antioxidant activity reinforces its potential for application in functional foods, especially when considering its origin as agro‐industrial waste.

WPY exhibited lower chroma values than DPY (Table 5), indicating reduced color intensity in the yogurt matrix. The coloration of yogurt is strongly influenced by the presence of phenolic compounds, which can interact with milk proteins and be affected by the system pH. Ye *et al*.^46^ reported that the addition of polyphenols may alter chromaticity through such interactions.

**Table 5:** Evaluation on the L*, a*, b* scale of the color of yogurts with and without peanut skin extract microparticles

Yogurt	L*	a*	b*
DPY	81.07 ± 2.72c	3.18 ± 0.35a	14.06 ± 0.74a
WPY	82.99 ± 2.46b	1.25 ± 0.69b	11.01 ± 1.38b
Without microparticles	87.95 ± 0.13a	−1.92 ± 0.01c	6.94 ± 0.02c

*Note:* Results expressed as mean ± standard deviation, *n* = 9; means followed by different lowercase letters differ statistically at the 5% error level by Tukey's test (*P* ≤ 0.05). DPY, Dry particles yogurt. WPY, Wet particles yogurt.

**Figure 5:**
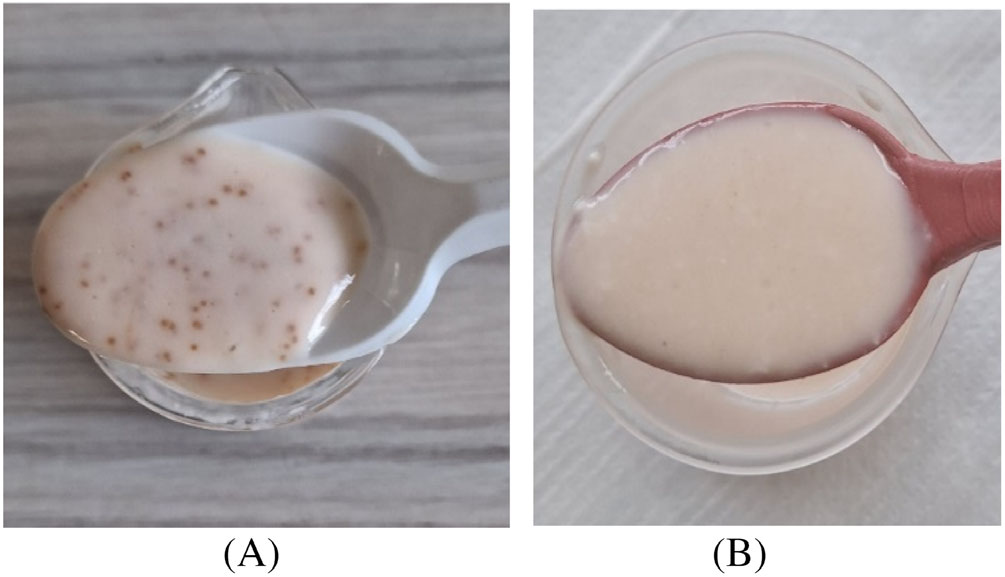
(A) Yogurt with 10% wet microparticles (WPY) and (B) yogurt with 0.6% dry microparticles (DPY).

In the present study, the lower chromaticity observed in WPY compared with DPY (Fig. 5) suggests that the encapsulation method influenced pigment dispersion within the dairy matrix. Microparticles produced by ionic gelation appeared to disperse within the yogurt mass in a way that reduced the overall color expression of the product. Nevertheless, further studies should assess sensory stability during storage as well as the bioavailability of phenolic compounds after consumption to confirm their effectiveness in the development of innovative dairy products.

Color analysis indicated that yogurts containing peanut skin extract exhibited slightly yellowish tones, with values of L* = 81.07, a* = 3.18, and b* = 14.06 for DPY, and L* = 82.99, a* = 1.25, and b* = 11.01 for WPY. In comparison, studies using *Hibiscus sabdariffa* reported greater red intensity, whereas research involving *Renealmia alpinia* described a more pronounced yellowish coloration. The milder chromatic effect observed with peanut skin extract may be advantageous in applications where maintaining the traditional appearance of yogurt is desirable without substantially altering its color. Additionally, Moura *et al*.^49^ demonstrated that the incorporation of hibiscus extract combined with microencapsulated hibiscus can enhance both the visual and functional appeal of yogurt while maintaining good sensory acceptance.

Overall, the results indicate that encapsulated peanut skin extract, in both dry and wet forms, provides a relevant source of phenolic compounds and antioxidant activity while producing only subtle changes in yogurt color. Considering that peanut skin is an agro‐industrial byproduct, its incorporation into dairy formulations represents a sustainable and promising strategy for the development of functional dairy products.

Table 6 presents the centesimal composition of yogurts with peanut skin extract microparticles in dry (DP) and wet (WP) form.

**Table 6:** Proximate composition of yogurts with and without peanut skin extract microparticles

Determination	DPY	WPY	Without microparticles
Moisture and volatiles (g kg^−1^)	89.93 ± 0.01a	90.67 ± 0.25a	88.46 ± 0.05a
Ash (g kg^−1^)	0.81 ± 0.00a	0.80 ± 0.00a	0.93 ± 0.00a
Total fats (g kg^−1^)	ND < 0.10d	ND < 0.10d	0.15 ± 0.02c
Protein (N × 6.38) (g kg^−1^)	3.37 ± 0.02a	3.19 ± 0.06a	4.36 ± 0.04
Total carbohydrates (g kg^−1^)	5.89b	5.34b	6.10b
Total calories (kcal kg^−1^)	37a	34a	38a

*Note:* Results expressed as mean ± standard deviation, *n* = 9; means followed by different lowercase letters differ statistically at the 5% error level by Tukey's test (*P* ≤ 0.05). DPY, Dry particles yogurt. WPY, Wet particles yogurt.

The moisture content did not show a significant difference between WPY (90.67%) compared to DPY (89.93%), which may be related to water retention in WP. A difference in moisture content could have influenced the texture of the final product, as observed in studies on the incorporation of ingredients rich in bioactive compounds into yogurts.

The protein content remained similar between the samples (DPY: 3.37 g kg^−1^; WPY: 3.19 g kg^−1^), which indicates that the addition of peanut skin extract microparticles did not significantly alter this component. This is relevant since protein stability directly influences the texture and sensory acceptance of yogurts.

The ash content also did not show a significant difference between DPY (0.81 g kg^−1^) and WPY (0.80 g kg^−1^), which suggests that the addition of peanut skin extract microparticles did not affect the amount of minerals present in the final product. Similar results were found in yogurts enriched with plant extracts, where the milk matrix maintained its mineral composition even after the addition of new ingredients.^47^

Carbohydrates showed no significant difference between the formulations, being 5.89 g kg^−1^ in DPY and 5.34 g kg^−1^ in WPY. The lower amount in WPY may be related to the greater retention of moisture, resulting in solids fraction dilution. This variation was also reflected in the energy value, being 37 kcal kg^−1^ in DPY and 34 kcal kg^−1^ in WPY. These results suggest that the addition of peanut skin extract microparticles did not compromise the nutritional composition of yogurt, which may be advantageous for the development of functional dairy products enriched with bioactive compounds.

According to Normative Instruction 46/2007,^41^ yogurts must have at least 2.9 g of milk protein per 100 g, and yogurts in this study were above this minimum limit due to the addition of 2% to 3.3% of skimmed milk powder. According to this same regulation, yogurts can also be classified according to their fat content as skimmed (maximum of 0.5 g of milk fat kg^−1^), partially skimmed (maximum of 2.9 g of milk fat kg^−1^) and full‐fat (minimum of 3 g of milk fat kg^−1^), with the fat content of yogurts in this study (ND < 0.10) classifying them as skimmed.

Regarding the microbiological characteristics, the identity and quality standard (IN 46/2007, BRAZIL)^44^ for yogurt establish that the minimum total lactic acid bacteria (LAB) count must be 107 CFU g^−1^. Samples of traditional yogurt without added extract showed LAB counts of 2.8 × 10^9^ and 3.1 × 10^9^ CFU g^−1^ after manufacturing and after 42 days of storage, respectively. Samples of traditional yogurt with extract showed counts of 1.9 × 10^9^ and 3.3 × 10^9^ CFU g^−1^ after manufacturing and after 42 days of storage, respectively. Thus, all samples produced were above the minimum count required even after 45 days of storage. This result is important because it indicates that the addition of peanut shell extract did not impair the viability of the LAB present and, therefore, does not alter this relevant characteristic of the fermented product.

All yogurt samples produced in this study were below the detection limit of the method for total coliforms, thermotolerant coliforms, and molds and yeasts, indicating adequate hygienic and sanitary conditions of the raw materials, the environment, and the handlers.

### Stability study of yogurts with microparticles

#### Quantification of phenolic compounds in yogurt with microencapsulated peanut skin extract

This study monitored total phenolic compounds in yogurts with peanut skin extract (wet and dry microparticles), revealing fluctuations over 42 days of storage.

DPY showed an initial phenolic content of 183.85 mg (100 g)^−1^, remaining stable until day 7, followed by a decrease between days 15 and 29, likely due to oxidation or interactions with milk proteins. After day 36, the phenolic content increased, reaching 195.73 mg (100 g)^−1^ on day 42, possibly reflecting a gradual release of bioactive compounds.

WPY initially had lower phenolic concentration (160.1 mg (100 g)^−1^), which increased until day 15 and slightly decreased by day 29, suggesting partial degradation or complexation of compounds. From day 36 onward, stabilization and a slight increase were observed (172.66 mg (100 g)^−1^ on day 42), indicating controlled phenolics release from the wet particles over time.

Silva *et al*.^50^ reported 640.69 mgGAE (100 g)^−1^ of phenolic compounds in skimmed yogurt with prickly pear jelly, a value higher than that found by Leite^51^ in yogurt with 25% *açaí* pulp (117.84 mgGAE (100 g)^−1^). Pádua *et al*.^52^ obtained 387 mgGAE (100 g)^−1^ in banana‐flavored yogurt with *jabuticaba* peel flour, while Moura *et al*.^47^ observed only 5.45 ± 0.20 mgGAE (100 g)^−1^ in yogurt containing *Hibiscus sabdariffa L*. particles produced by ionic gelation. The higher concentration found in this study highlights the functional potential of the product. Moreover, the use of peanut skins represents an innovative strategy for valorizing an agro‐industrial byproduct, adding value to a material that would otherwise be discarded.

According to Moura *et al*.,^47^ some components such as yogurt proteins have the effect of interfering in this analysis, producing blue color. Total polyphenol content results achieved by the Folin–Ciocalteu method can only be reliable after the removal of such interfering compounds, with such extraction being possible by sample purification only. With respect to yogurt with particles, separation of particles from yogurt mass was possible, thus enabling better quantification of total polyphenols arising from the hibiscus extract present in particles.

#### Quantification of antioxidant activity (DPPH) in yogurt with microencapsulated peanut skin extract

**Figure 6:**
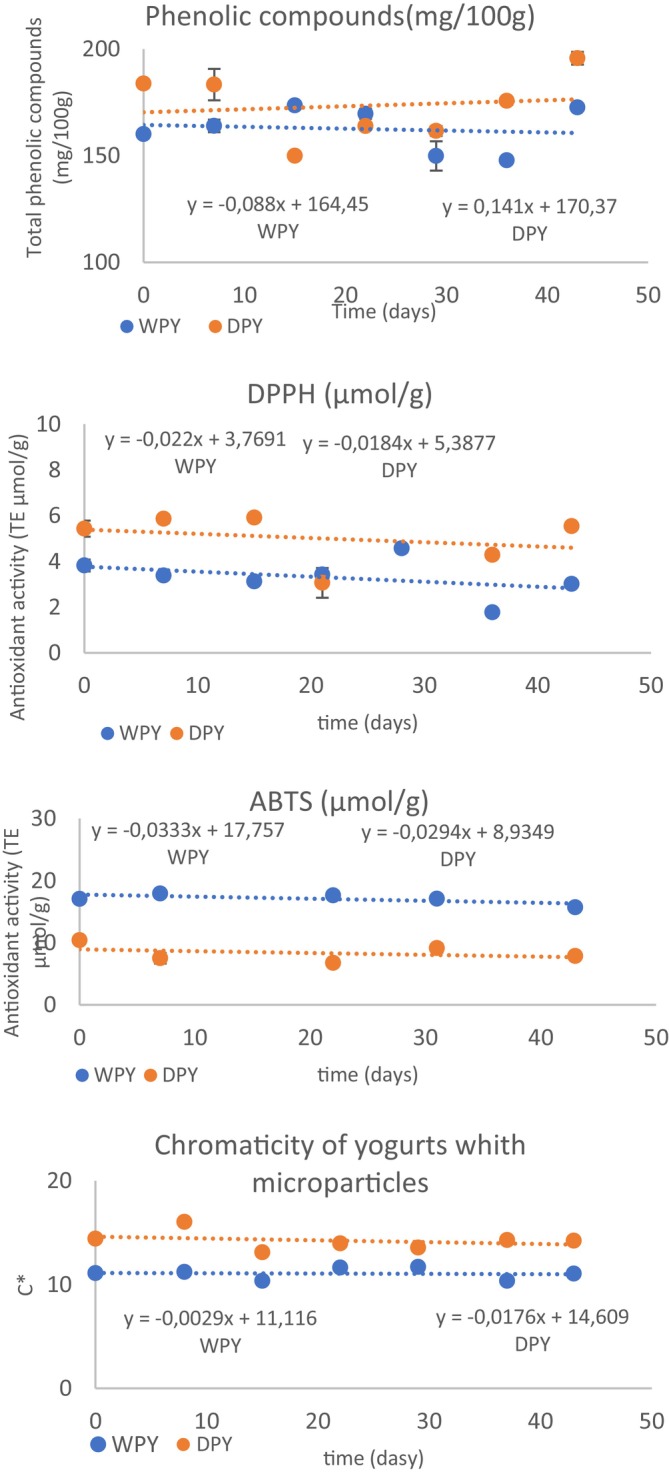
Stability of yogurts with wet and dry peanut skin microparticles during storage.

Figure 6 shows the results of antioxidant activity measured by DPPH during storage of yogurt with added wet (WP) and dry (DP) particles of peanut skin extract. In DPY, the initial antioxidant activity was 5.43 μmol TE g^−1^ and showed 5.54 μmol after 42 days of storage. On the other hand, WPY showed a different behavior. The initial antioxidant activity was 3.82 μmol TE g^−1^, registering a peak of 4.57 μmol TE g^−1^ on the day 28, followed by a sharp decrease to 3.01 TE g^−1^ on day 42. This gradual decrease indicates a loss of antioxidant capacity, probably due to the degradation of phenolic compounds over time.

The results obtained suggest that the encapsulation method directly impacts the stability of bioactive compounds in the food system. WP, formed by ionic gelation, demonstrated a greater capacity for preserving antioxidant activity throughout storage.

#### Quantification of antioxidant activity (ABTS) in yogurt with microencapsulated peanut shell extract

The antioxidant activity of yogurts containing wet (WP) and dry (DP) peanut skin extract particles was monitored over 42 days using the ABTS method (Fig. 6). WPY exhibited an initial activity of 17.04 μmol TE g^−1^ decreasing slightly to 15.71 μmol TE g^−1^ by day 42, indicating good stability likely due to protection from the ionic gelation matrix. In contrast, DPY started at 10.41 μmol TE g^−1^ and dropped sharply to 7.87 μmol TE g^−1^ with greater fluctuations during storage, reflecting the lower protective capacity of spray‐dried particles against oxidative degradation.

Vargas *et al*.^53^ demonstrated that spray drying effectively preserves the bioactive compounds and antioxidant activity of *açaí* pulp, protecting phenolics, flavonoids, anthocyanins, and ascorbic acid from environmental degradation and extending the shelf‐life. Similarly, Santana^54^ reported that ionic gelation enhances the stability and durability of microencapsulated *camu‐camu* juice, allowing a gradual release of bioactive compounds and maintaining higher antioxidant activity.

The yogurts in this study exhibited antioxidant capacities of 5.43 μmol TE g^−1^ (DPY) and 3.82 μmol TE g^−1^ (WPY), indicating that peanut skin extract enhances the product functional properties. Previous studies reported lower values, such as that of Moura *et al*.^47^ with *Hibiscus sabdariffa* L. microparticles (0.22 ± 0.02 TE μmol TE g^−1^) and Jimenez‐Gonzalez *et al*.^48^ with *Renealmia alpinia* spray‐dried microparticles (1 mg TE (100 g)^−1^). However, differences in methodology and units limit a direct comparison. The notable antioxidant activity observed here highlights the potential of peanut skin, an agro‐industrial byproduct, to be used in functional foods.

#### Instrumental color evaluation in yogurts with microencapsulated peanut skin extract

Color is a key attribute influencing consumer acceptance of fermented dairy products, particularly yogurts enriched with bioactive compounds. The color parameters (L*, a*, b*, and Δ*E*) were monitored for both yogurt formulations during 42 days of storage (Table 7). Initially, WPY showed higher L* values (82.99) than DPY (81.07), indicating a lighter appearance. This difference may be associated with the dispersion behavior of the particles: dry particles tend to disperse more uniformly within the yogurt matrix, contributing to a darker appearance, whereas wet particles remain less integrated, resulting in a lighter product.

**Table 7:** Results of monitoring color parameters of yogurt with microencapsulated peanut skin extract for a period of 42 days

	WPY				DPY			
Time (days)	L*	a*	b*	Δ*E*	L*	a*	b*	Δ*E*
0	82.99ab ± 2.46	1.25b ± 0.69	11.01a ± 1.38		81.07ab ± 2.72	3.18bc ± 0.35	14.06bc ± 0.74	
8	85.03a ± 1.42	1.31b ± 0.89	11.11a ± 2.05	2.08	81.95ab ± 2.48	4.24a ± 0.54	15.45a ± 0.73	1.91
15	80.44b ± 2.86	1.38ab ± 0.26	10.25b ± 0.58	3.56	79.24b ± 1.66	2.80c ± 0.12	12.80d ± 0.32	2.54
22	85.50a ± 2.44	1.81ab ± 0.46	11.48ab ±1.05	3.41	81.21ab ± 3.18	3.05bc ± 0.20	13.62bcd ±0.71	0.11
29	84.08a ± 2.19	1.64ab ± 0.32	11.57ab ± 0.77	0.83	80.48ab ± 2.43	2.97bc ± 0.10	13.22 cd ± 0.47	0.55
37	82.59ab ±2.37	1.35ab ± 0.51	10.26b ± 0.87	0.37	79.99ab ± 1.33	3.18bc ± 0.59	13.92bc ± 1.06	0.58
42	83.44a ± 1.58	1.46a ± 0.55	10.95a ± 1.14	0.12	80.66a ± 1.36	3.24b ± 0.32	13.85b ± 0.65	0.11

*Note:* Results expressed as mean ± standard deviation, *n* = 3; means followed by different lowercase letters statistically decreased at the 5% error level by Tukey's test (*P* ≤ 0.05). DPY, Dry particles yogurt. WPY, Wet particles yogurt.

The a* parameter, which indicates redness, was lower in WPY and higher in DPY. The greater red intensity observed in DPY may also be related to a more homogeneous distribution of dry particles within the yogurt matrix. Similarly, the b* parameter (yellowness) was higher in DPY than in WPY, suggesting that dry particles contributed to a slightly more yellowish tone in the final product. Despite these differences, the total color difference (Δ*E*) remained below 3.56 for both yogurts throughout storage, indicating overall color stability. Chroma (C*), presented in Fig. 6, reflects color saturation. Higher values were observed in DPY, particularly during the initial storage period (16.02), compared with WPY (11.68), reinforcing the tendency of dry particles to intensify color in the yogurt matrix.

Overall, yogurts containing peanut skin extract exhibited slightly yellowish tones, with values of L* = 81.07, a* = 3.18, and b* = 14.06 for DPY, and L* = 82.99, a* = 1.25, and b* = 11.01 for WPY. In comparison, studies with *Hibiscus sabdariffa* reported greater red intensity, while research using *Renealmia alpinia* showed more pronounced yellowish coloration. The milder chromatic effect of peanut skin extract may be advantageous in applications where maintaining the traditional appearance of yogurt is desirable without markedly altering the product color. Additionally, Moura *et al*. demonstrated that the incorporation of hibiscus extract combined with microencapsulated hibiscus can enhance both the visual and functional appeal of yogurt, while maintaining good sensory acceptance.

#### Evaluation of pH and acidity in yogurts with microencapsulated peanut skin extract

Over 42 days of storage, yogurts with wet (WPY) and dry (DPY) peanut skin microparticles showed significant variations in pH and titratable acidity (Table 8). pH values fluctuated between 4.08 and 4.49, remaining within the typical range for fermented dairy products, with significant differences between formulations at each time point (*P* < 0.05). Initially, DPY had a higher pH (4.32) than WPY (4.20), but by day 7, that of WPY surpassed DPY (4.28 *versus* 4.24). From day 14, both showed a pH decline, reaching a minimum in DPY at day 21 (4.08), followed by a non‐significant increase from day 28 to day 42, with DPY reaching the highest value (4.49). These fluctuations may result from degradation of acids, substrate consumption, or release of buffering compounds from the microparticles.

**Table 8:** Results of monitoring pH and titratable acidity of yogurts with microencapsulated peanut skin extract for a period of 42 days

	pH		Titratable acidity	
Time (days)	WPY	DPY	WPY	DPY
0	4.20c ± 0.00	4.32b ± 0.00	0.529a ± 0.003	0.540a ± 0.001
7	4.28b ± 0.00	4.24c ± 0.00	0.527a ± 0.011	0.550a ± 0.008
14	4.20c ± 0.01	4.13d ± 0.02	0.499b ± 0.008	0.524b ± 0.003
21	4.10d ± 0.00	4.08e ± 0.00	0.539a ± 0.008	0.542a ± 0.005
28	4.39a ± 0.00	4.49a ± 0.00	0.537a ± 0.007	0.553a ± 0.003
42	4.40a ± 0.00	4.50a ± 0.00	0.538a ± 0.007	0.553a ± 0.002

*Note:* Results expressed as mean ± standard deviation, *n* = 3; means followed by different lowercase letters statistically decreased at the 5% error level by Tukey's test (*P* ≤ 0.05). DPY, Dry particles yogurt. WPY, Wet particles yogurt.

Titratable acidity remained largely stable (0.499–0.553%) over storage with a minor difference at day 14, indicating controlled microbial activity and effective buffering from the encapsulation systems. Variations in pH and acidity may be influenced by the phenolic compounds in peanut skin extract and the encapsulation form, which affects the gradual release of bioactive compounds and their interaction with the yogurt matrix, highlighting the importance of incorporation method for product stability.

## CONCLUSIONS

After studying two different microencapsulation methods for protecting peanut skin extract, we concluded that the ionic gelation method followed by the absorption technique (WP) demonstrated technical viability, showing better particle coloration and a significantly higher content of phenolic compounds. This indicates that the method can be tested for application in industrialized products, especially those with higher moisture content, such as dairy products and beverages. The microparticles obtained by spray drying (DP) also showed a significant content of phenolic compounds and can probably be better applied in industrialized products with low moisture content, such as confectionery (candies, chocolates). The stability of the microparticles was observed when stored under controlled temperature, but future studies will still be necessary to prove their stability when applied to other manufactured products, since their interaction with other ingredients may affect bioactive compound stability.

The results of this study demonstrated that the addition of peanut skin extract microparticles to yogurt, both in dry form (DP) and in wet form (WP), did not compromise the centesimal composition of the product, maintaining similar levels of moisture, ash, proteins, and fats. Furthermore, the presence of microparticles did not negatively affect the population of LAB, ensuring the microbiological viability of the yogurt. Regarding the bioactive composition, yogurts containing WP had a slightly higher content of phenolic compounds compared to DP, although without a statistically significant difference. The antioxidant capacity was similar between both formulations, indicating that the retention of bioactive compounds was preserved. The color analysis revealed that the addition of microparticles influenced the product chromaticity, especially in WPY, which presented lower total color intensity. This effect may be associated with interactions between phenolic compounds and proteins of the milk matrix, in addition to variations in the system pH, as already described in the literature for dairy products enriched with polyphenols. The stability study of WPY and DPY products showed that antioxidant activity, color, and acidity were maintained for at least 30 days of storage. Sensory tests with a trained team of tasters are necessary to validate the results, especially regarding yogurt flavor.

The findings demonstrate the feasibility of transforming peanut skin into a value‐added functional ingredient for dairy products. The developed systems show strong potential for application in functional yogurts, antioxidant‐enriched dairy formulations, and clean‐label products, supporting sustainable waste valorization and circular economy strategies. Larger‐scale testing and longer‐duration tests are needed to confirm the viability of industrial‐scale application. Overall, this study provides an innovative, scalable, and environmentally responsible approach for enhancing the nutritional and functional properties of yogurt while reducing agro‐industrial waste.

## CONFLICT OF INTEREST

The authors declare that they have no known competing financial interests or personal relationships that could have appeared to influence the work reported in this paper.

## ETHICS STATEMENT

The authors declare that all experiments in this study were conducted according to the established ethical guidelines.

## AUTHOR CONTRIBUTIONS

**Ana Carolina Tucumantel Ribeiro:** Writing – review & editing, Writing – original draft, Investigation, Formal analysis, Data curation. **Victor Waldemar Pinto** and **Jaqueline LS Cavalcante:** Data curation, Formal analysis. **Natália Almeida Costa** and **Bárbara M Lepaus:** Formal analysis. **Juliana A Macedo:** Investigation, Funding acquisition. **Patrícia Blumer ZR Sá:** Writing – review & editing, Methodology, Conceptualization. **Sílvia Cristina Sobottka Rolim de Moura**: Writing – review & editing, Methodology, Conceptualization, Supervision, Project administration.

## Data Availability

The data that support the findings of this study are available on request from the corresponding author. The data are not publicly available due to privacy or ethical restrictions.
